# The Impact of the COVID-19 Pandemic on Surgical Activities: A Single-Center Experience and Literature Review

**DOI:** 10.7759/cureus.30785

**Published:** 2022-10-27

**Authors:** Adam Mylonakis, Areti Kalfoutzou, Andreas Panagakis, Markos Despotidis, John Yfantopoulos

**Affiliations:** 1 First Department of Surgery, Laiko General Hospital, National and Kapodistrian University of Athens, Athens, GRC; 2 Oncology Department, 251 Air Force General Hospital, Athens, GRC; 3 Health Economics, University of Athens, Athens, GRC

**Keywords:** surgical oncology, emergency surgery, elective surgery, surgical activity, covid-19

## Abstract

Aim

The aim of this article is to investigate the effect of the coronavirus disease 2019 (COVID-19) pandemic on our surgical department, which is situated in Athens, Greece, as well as to review published literature on the COVID-19 pandemic's impact on surgical activities in our department.

Material and methods

We retrospectively reviewed the surgical procedures that were performed in the surgical department of a tertiary University hospital in Athens, Greece, before and during the pandemic. Furthermore, we performed a literature review evaluating articles on surgical activity and COVID-19 published from the beginning of the pandemic up until the January of 2022 on the PubMed database.

Results

In total, 894 patients were included in the study. Of those, 264 (29.5%) underwent surgery during the control period and 630 (70.5%) in the pandemic period. Overall, we performed 20.5% fewer surgeries in the post-sanitary period. In particular, elective surgeries decreased on average by 23.9%, emergency procedures decreased by 8.9%, and oncology surgeries increased by an average of 6.4% after the year 2020. Concerning the review of literature, 51 studies were selected for this review. According to them, the main effect of the pandemic on the surgical sector was reflected in the reduction of total surgeries, mainly due to the postponement of elective surgical procedures, which showed a median reduction of 54% compared to the pre-COVID-19 period. A smaller decrease was observed in the number of emergency and oncological surgeries.

Conclusions

Reduced surgical activity during the pandemic, due to the health measures imposed, requires courageous corrective interventions to avoid its adverse effects, such as disease progression, increased treatment costs, reduced quality of life, and ultimately the survival of the patients.

## Introduction

Following a series of cases of pneumonia of unknown etiology in Hubei province, China, Severe Acute Respiratory Syndrome Coronavirus 2 (SARS-CoV-2) was isolated as the cause of coronavirus disease 2019 (COVID-19) [1]. The virus spread rapidly worldwide and led the World Health Organization to declare the novel coronavirus (COVID-19) outbreak a global pandemic on 11 March 2020 [2].

COVID-19 has proven to be a complex entity, having a high rate of transmissibility, numerous mutations, and causing multisystem disorder [3]. The often-exponential spread of the pandemic has highlighted the greatest challenge: the increased need for health services in health systems with finite resources. This mismatch of resources and needs has led to not only radical changes in the supply of health services but also in socio-economic relationships in general.

The impact of the pandemic on the surgical sector is multifaceted and concerns the surgical staff and practice, the risk of transmission between patients and medical personnel, and lastly, medical education. In response to these challenges, the following guidelines were issued for the safe practice of surgical practice [4,5]: preparation of a surgical case management plan in case of deterioration of the epidemiological picture, postponement of elective surgeries, conversion of operating rooms into intensive care units, ensuring a safe response to surgical emergencies, training of staff in the use of personal protective equipment and protocols, minimizing staff exposure, early identification, and treatment of COVID-19 infections in surgical patients and development of septic operating rooms for the surgical treatment of positive cases.

The aim of this article is to investigate how the COVID-19 pandemic outbreak affected surgical activities in our surgical department as reflected in the number and type of surgical procedures performed before and during the pandemic. In addition, we reviewed published literature concerning the COVID-19 pandemic's impact on surgical activities worldwide.

## Materials and methods

The electronic medical records of the patients included in this survey were collected from the First Department of Surgery of "Laikon" General Hospital, a tertiary University hospital in Athens, Greece. Patients' records were retrospectively reviewed in regard to their age, gender, and reason for admission/surgical procedure performed. We compared two time periods, i.e., before the pandemic from January until April 2019 (control group), and during the pandemic from January until April of the years 2020-2022 (pandemic period). Exclusion criteria were all patients that did not undergo a surgical procedure under general anaesthesia. This study was exempted from Hospital Review Board as it involved the collection and study of existing recorded data so that subjects cannot be identified, directly or through identifiers linked to the subjects.

The literature review was conducted in accordance with the Preferred Reporting Items for Systematic Reviews and Meta-Analyses (PRISMA) guidelines. A systematic web-based search using Medline was performed reviewing literature published between 1st December 2019 and 31st January 2022. We limited the search to English language articles using two sets of keywords: (COVID-19) AND ({surgery} OR {surgical department}) and (COVID-19) AND ({operations} OR {surgical volume} OR {emergency procedure}).

After screening and identification of the relevant studies, detailed information was extracted from the full-text articles regarding the following: type of study, number of patients, country of origin, medical specialty, change in the volume of the total, elective, emergency, and oncological surgical procedures.

Statistical analysis was performed using the statistical package SPSS, version 25.0 (IBM Corp., Armonk, NY). The normality of numerical data distribution was tested using the Kolmogorov-Smirnov test. To examine the statistical difference, we used the independent t-test for continuous variables and the Chi-square test for categorical variables. The level of statistical significance was set at p<0.05 (two-tailed).

## Results

In total, 894 patients were included in the study. Of those, 264 (29.5%) underwent surgery during the control period and 630 (70.5%) in the pandemic period. No significant differences in mean age (62.1 years in the COVID-19 cohort vs 63.4 years in the control group, p=0.146) or gender distribution (58% males within the COVID-19 cohort vs 52.1% among the control cohort, p= 0.107) were noted (Table 1).

**Table 1:** Demographic data of patients operated in the First Department of Surgery, National and Kapodistrian University of Athens, Laikon General Hospital

		Control Period	Pandemic Period	p-value
Number of patients		264	630	
Gender (%)	Female	111 (42%)	302 (47.9%)	P= 0.107
	Male	153 (58%)	328 (52.1%)	
Age, years mean ±SD		63.4±13.7	62.1±11.5	P= 0.146

Overall, we performed 20.5% fewer surgeries in the post-sanitary period. In particular, elective surgeries showed a decrease of 23.9%, while emergency procedures were less affected, with an average decrease of 8.9%. Oncology surgeries did not show a decrease but instead, increased by an average of 6.4% after the year 2020. From the statistical study of the above sample, there is no statistically significant change between the control and the pandemic cohort, except for the category of elective surgical operations (p= 0.044) (Table 2).

**Table 2:** Surgical operations performed in the First Department of Surgery, National and Kapodistrian University of Athens, Laikon General Hospital

	Jan- April 2019	Jan- April 2020	Jan- April 2021	Jan- April 2022	% Mean change 2020-2022 v 2019	p-value (Calculated based on monthly operations)
Total operations	264	231	186	213	-20.5%	0.052
Elective operations	204	177	135	157	-23.9%	0.044
Emergency operations	60	54	54	56	-8.9%	0.592
Oncological operations	94	129	84	87	+6.40%	0.739

**Figure 1:**
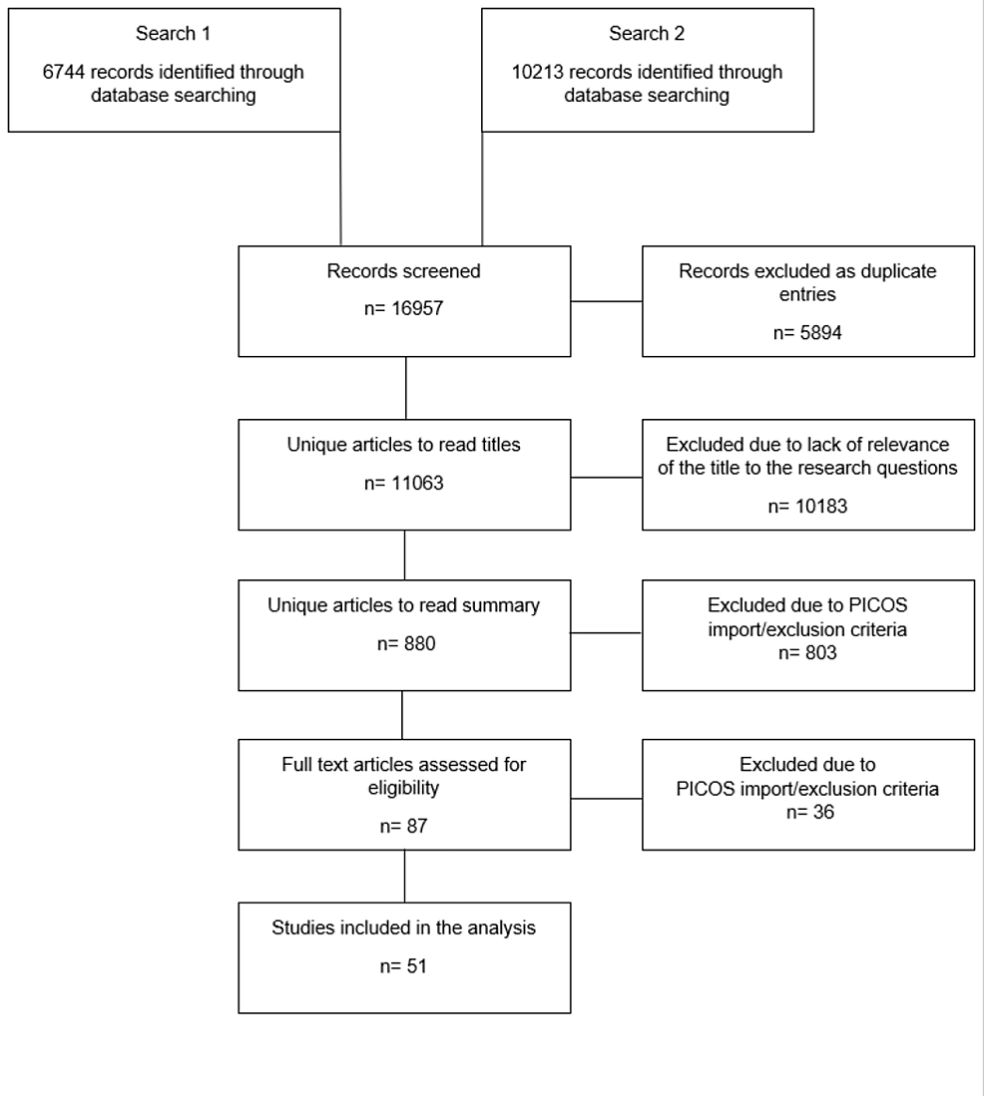
PRISMA flowchart PRISMA: Preferred Reporting Items for Systematic Reviews and Meta-Analyses

The literature review search produced 11063 unique PubMed results. Articles matching our selection criteria were 51 and were used for data collection [6-56] (Figure 1).

The majority of the researchers collected samples from a surgical department of a hospital, while 14 researchers drew data from national databases. With regard to the surgical specialty, general surgery was the most represented specialty, with 13 of the articles studying the whole surgical sector (Table 3).

**Table 3:** Studies included in the review by sample size and surgical specialty ENT: Ear, Nose, Throat

	n	%
By sample size		
Surgical Department	28	55%
Hospital	5	10%
Series of Hospitals	4	8%
National Registry	14	27%
By Surgical Specialty		
Vascular Surgery	4	8%
General Surgery	18	35%
Gynecology	3	6%
Cardiac Surgery	3	6%
Neurosurgery	2	4%
Orthopaedics	3	6%
Urology	2	4%
Surgical Oncology	2	4%
ENT	1	2%
Surgical Sector	13	25%

We observed a decrease in the total surgical activity after the pandemic outbreak compared to the pre-COVID-19 period in 23 of the 24 studies, The reduction was significant in all surgical specialties and the median decrease was 50.7% (Table 4).

**Table 4:** Change in the total volume of operations ENT: Ear, Nose, Throat

Author	Specialization-Sector	Change in the total volume of operations
Dallan et al. 2021 [14]	Cardiac surgery	-65.8%
Mejia et al. 2021 [34]	Cardiac surgery	-62%
Ralli et al. 2020 [45]	ENT	-50.7%
Alsaoudi et al. 2020 [7]	General Surgery	42.5%
Kreis et al. 2021 [26]	General Surgery	-11.3%
Yoon et al. 2021 [55]	General Surgery	-74%
Piketty et al. 2021 [44]	Gynaecology	-65%
Spurlin et al. 2021 [50]	Gynaecology	-79.3%
Raneri et al. 2020 [46]	Neurosurgery	-30%
Wali et al. 2021 [54]	Neurosurgery	Unchanged
Koch et al. 2021 [25]	Orthopaedics	-35%
Magnusson et al. 2021 [30]	Orthopaedics	-70%
Shih et al. 2021 [49]	Orthopaedics	-29%
Vissio et al. 2021 [53]	Surgical Oncology	-11.8%
Dias et al. 2021 [17]	Vascular Surgery	-64.7%
Abram et al. 2021 [6]	Surgical Sector	-65.4%
Di Marzo et al. 2020 [16]	Surgical Sector	-68%
Farid et al. 2020 [56]	Surgical Sector	-80%
Gomez et al. 2021 [22]	Surgical Sector	-78 to -83%
Ikeda et al. 2021 [23]	Surgical Sector	-10 to-15%
Luizeti et al. 2021 [29]	Surgical Sector	-14.9%
Mazahreh et al. 2020 [32]	Surgical Sector	-88.7%
Miyawaki et al. 2021 [36]	Surgical Sector	-9.4%
Rose et al. 2021 [47]	Surgical Sector	-75%

Furthermore, we reported a decrease in elective surgical procedures in all the articles studying this topic. The median decrease was 54% and exceeded 90% in certain cases (Table 5).

**Table 5:** Change in volume of elective operations

Author	Specialization-Sector	Change in volume of elective operations
Salenger et al. 2020 [48]	Cardiac surgery	-54%
Metelmann et al. 2020 [35]	General Surgery	-34.9%
Kreis et al. 2021 [26]	General Surgery	-85%
Raneri et al. 2020 [46]	Neurosurgery	-34%
Shih et al. 2021 [49]	Orthopaedic Surgery	-31%
Leung et al. 2021 [28]	Vascular Surgery	-42.8%
Dias et al. 2021 [17]	Vascular Surgery	-87%
Abram et al. 2021 [6]	Surgical Sector	-89%
Mazahreh et al. 2020 [32]	Surgical Sector	-92.8%
Gomez et al. 2021 [22]	Surgical Sector	-36 to -49%
Sutherland et al. 2020 [51]	Surgical Sector	-32.6%
Luizeti et al. 2021 [29]	Surgical Sector	-34.8%
Di Marzo et al. 2020 [16]	Surgical Sector	-75%

With regard to emergency surgical procedures, the data of the literature review indicate a reduction in 18 of the 22 relevant studies, with a median decrease of 30% (Table 6).

**Table 6:** Change in volume of emergency operations

Author	Specialization-Sector	Change in volume of emergency operations
Karlafti et al. 2021 [24]	General Surgery	-51%
D'Urbano et al. 2020 [18]	General Surgery	-41.3%
Tartaglia et al. 2020 [52]	General Surgery	-42.8%
Malik et al. 2021 [31]	General Surgery	18%
O'Connell et al. 2021 [39]	General Surgery	-25.4%
Castoldi et al. 2021 [12]	General Surgery	-60%
Kreis et al. 2021 [26]	General Surgery	52.3%
Palisi et al. 2020 [42]	General Surgery	Unchanged
Patriti et al. 2020 [43]	General Surgery	-86%
Piketty et al. 2021 [44]	Gynaecology	-64%
Spurlin et al. 2021 [50]	Gynaecology	Unchanged
Raneri et al. 2020 [46]	Neurosurgery	-23%
Shih et al. 2021 [49]	Orthopaedic	-13.3%
Ralli et al. 2020 [45]	Otolaryngology	-60%
Motterle et al. 2021 [37]	Urology	-52%
Correia et al. 2020 [13]	Vascular Surgery	Unchanged
Leung et al. 2021 [28]	Vascular Surgery	-31.6%
Dias et al. 2021 [17]	Vascular Surgery	Unchanged
Abram et al. 2021 [6]	Surgical Department	-35.3%
Mazahreh et al. 2020 [32]	Surgical Department	-60.4%
Luizeti et al. 2021 [29]	Surgical Department	-1.1%
Di Marzo et al. 2020 [16]	Surgical Department	-30%

Concerning oncological surgery, we noticed a statistically significant decrease in surgical activity in three out of nine articles, reaching up to a reduction of 56% in a general surgery clinic in Parma, Italy [21] (Table 7).

**Table 7:** Change in volume of oncological operations

Author	Specialization-Sector	Change in volume of oncological operations
Giuffrida et al. 2021 [21]	General Surgery	-56%
Nogami et al. 2022 [38]	Gynaecology	-3.9%
Ralli et al. 2020 [45]	Otolaryngology	Unchanged
Vissio et al. 2021 [53]	Surgical Oncology	-11.8%
Kuitunen et al. 2021 [27]	Surgical Oncology	Unchanged
Abram et al. 2021 [6]	Surgical Department	-47.8%
de Pelsemaeker et al. 2020 [15]	Surgical Department	Unchanged
Luizeti et al. 2021 [29]	Surgical Department	-5%
Okuno et al. 2021 [40]	Surgical Sector	Unchanged

## Discussion

In our surgical department, we observed an average decrease of 20.5% in the total number of surgical procedures performed after the COVID-19 outbreak, mainly due to the postponement of elective surgeries. Emergency cases were less affected and in regard to oncological procedures, we noted an average increase of 6.4%. This data aligns with the current literature suggesting that the surgical sector worldwide prioritized emergency and oncological over elective non-life-threatening cases.

COVID-19 is manifested mainly as an acute respiratory disease, which can be complicated by Acute Respiratory Distress Syndrome (ARDS) and multi-organ failure. Its global spread has put severe pressure on Health Systems worldwide, resulting, among other things, in the transfer of resources from the surgical sector to the medical units directly involved in the fight against the pandemic. The release of personnel and equipment from surgical units is not without negative effects, the results of which will be revealed over a horizon of years or even decades.

Following the recommendation to postpone non-life-threatening surgeries issued by WHO and major surgical colleges [57-60], a reduction of surgical operations for benign or non-life-threatening diseases was expected. It is worth noting that delaying the treatment of such conditions is not free of consequences, as it can lead to a worsening of the state of health, an increasing disability and a decrease in the working capacity of patients. These effects entail significant social costs, especially in low-middle-income countries, where costs related to the surgical condition can lead to impoverishment [61].

Similar to elective surgical procedures, the observed decrease in emergency surgical operations needs to be investigated. On the one hand, the change may be due to a real decrease in emergencies due to the recommendation to stay at home and avoid social activity. These measures have resulted in a shrinking number of sports [62], road accidents [63] injuries and the spread of communicable diseases [64] other than COVID-19.

At the same time, the issued guidelines to avoid unnecessary attendance at hospitals have led patients to seek late medical help possibly awaiting later stages of the disease before attending the emergency department [6,9,13,26,31,52,55]. Their aggravated condition was associated with increased complication rates [18,31,43] and mortality rate [14,17,24]. Taking into account the inherent inability of the patients to evaluate the severity of their condition, it is crucial to inform the population about the nature of emergency surgical conditions as well as to prepare for an outbreak of surgical cases during the periods of remission of the pandemic.

The management of oncological cases is a major priority of health systems, as a delay in their treatment has serious consequences for patients and society in general. Therefore, health authorities must ensure the proper and incessant function of screening, diagnosis and treatment of oncology patients. According to the research of Yun et al. [65] a postponement in the treatment of malignancy of more than one month is related to a worse prognosis for rectal and breast cancer in high-volume centers. In addition to increased mortality, delays also result in increased costs of care in the form of surgery and/or chemotherapy. More resources will be required if a patient presents with an emergency such as perforation, acute bleeding or gastrointestinal obstruction.

Another major issue, which is being overlooked with unknown long-term consequences is the training of medical staff. The reduction of surgical operations has a negative effect on the development of specialized surgeons, whose training requires a high number of surgeries, in order to acquire the necessary skills and techniques. Indicatively, Inzunza et al. [66] report a decrease of 61.7% in the total number of surgical procedures of the third-year surgical residents, possibly requiring an extension of the training programme of the affected trainees by at least one year.

Eventually with the expected recession of the pandemic, the health systems will face the accumulated volume of elective surgical cases which have been postponed since the beginning of 2020. Performing these operations will require funding from the surgical sector for an extended period of time. Indicatively, to carry out the postponed surgeries for a period of 12 weeks, with an increase of the basic activity by 20%, will require an average of 45 weeks (range 43-48 weeks) [61]. It is thus clear that a return to normal surgical practice and the clearance of the backlog of surgical work within a reasonable period of time requires major changes that go beyond the return of surgical personnel and equipment and extend to the reform of health systems expenditures.

The current study has some limitations. Firstly, it involved a single center in a tertiary referral hospital in Athens, Greece, so it does not reflect the pandemic impact across the country. Secondly, the literature review excluded data from non-English papers; Asian countries, which were particularly affected during the first wave of the pandemic, were represented in a limited number of surveys. In addition, it only included articles published in the PubMed database, thus missing data from other databases. Furthermore, the majority of studies included data for a specific time period, usually outbreaks, rather than for the entire pandemic, Finally, it is noted that the pandemic is still ongoing and despite the accumulated experience of the last two years, its course and impact on the health systems worldwide is still difficult to predict.

## Conclusions

The impact of the COVID-19 pandemic worldwide is apparent in all areas of surgical activity. It is reflected mainly in the reduction of elective surgical procedures and to a lesser extent in emergency and oncological cases, a trend that we also noticed in our surgical department. Reduced surgical activity has various adverse effects including disease progression, increased treatment costs, reduced quality of life, and ultimately the survival of the patients. 

In this setting, it is vital for all nations around the globe to take into account the malfunctions that occurred in the surgical sector during the pandemic to recognize the weaknesses of the health systems and draw up a plan based on the lessons learned to deal with a possible future epidemic disease.
